# Privacy Preserving RBF Kernel Support Vector Machine

**DOI:** 10.1155/2014/827371

**Published:** 2014-06-12

**Authors:** Haoran Li, Li Xiong, Lucila Ohno-Machado, Xiaoqian Jiang

**Affiliations:** ^1^Department of Mathematics & Computer Science, Emory University, Atlanta, GA 30322, USA; ^2^Division of Biomedical Informatics, UC San Diego, La Jolla, CA 92093, USA

## Abstract

Data sharing is challenging but important for healthcare research. Methods for privacy-preserving data dissemination based on the rigorous differential privacy standard have been developed but they did not consider the characteristics of biomedical data and make full use of the available information. This often results in too much noise in the final outputs. We hypothesized that this situation can be alleviated by leveraging a small portion of open-consented data to improve utility without sacrificing privacy. We developed a hybrid privacy-preserving differentially private support vector machine (SVM) model that uses public data and private data together. Our model leverages the RBF kernel and can handle nonlinearly separable cases. Experiments showed that this approach outperforms two baselines: (1) SVMs that only use public data, and (2) differentially private SVMs that are built from private data. Our method demonstrated very close performance metrics compared to nonprivate SVMs trained on the private data.

## 1. Introduction

Data sharing is important for accelerating scientific discoveries, especially when there are not enough local samples to test a hypothesis [1, 2]. However, medical data are sensitive as they essentially contain personal information and can reveal much about ethnicity, disease risk [3], and even family surnames [4]. To promote data sharing, it is important to develop privacy-preserving algorithms that respect data confidentiality and present data utility [5], especially when one wants to leverage cloud computing [6].

Privacy preserving data analysis and publishing [7, 8] have received considerable attention in recent years as a promising approach for sharing information while preserving data privacy. Differential privacy [9–11] has recently emerged as one of the strongest privacy guarantees for statistical data release [12–17]. A statistical aggregation or computation is DP (we shorten differentially private to DP) if the outcome is formally indistinguishable when run with and without any particular record in the dataset. The level of indistinguishability is quantified as a privacy parameter *ϵ*. A common mechanism to achieve differential privacy is the Laplace mechanism [18] which injects calibrated noise to a statistical measure determined by the privacy parameter *ϵ* and the sensitivity of the statistical measure influenced by the inclusion and exclusion of a record in the dataset. A lower privacy parameter requires larger noise to be added and provides a higher level of privacy.

**Figure 1:**
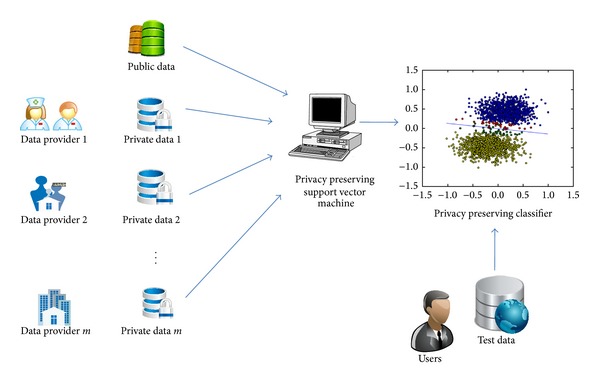
Biomedicine data sharing system. A small amount of public data and a large amount of private data are available for different data providers. A privacy preserving support vector machine can leverage both public and private data to maximize the classification accuracy under differential privacy. Then users can classify their test data via the released privacy preserving classifier.

General purpose algorithms for privacy protection (e.g., [19, 20]) often introduce too much perturbation error, which renders the resulting information useless for healthcare research. Our contribution is to leverage a small portion of open-consented data to maximally explore information that resides in the private data through a hybrid framework.  Figure 1 shows an example of an environment in this case. We recently published differentially private distributed logistic regression using public and private biomedical datasets [21], which demonstrated advantages over pure private or public models. However, logistic regression is a generalized linear model, which has limited flexibility in classifying complex patterns. In this paper, we sought to extend our previous effort to the more powerful, RBF-kernel based support vector machines.

The remainder of the paper is organized as follows.  Section 2 reviews background knowledge of differential privacy and SVM and RBF kernel.  Section 3 describes the framework and details for our hybrid SVM mechanism. Then, Section 4 contains an extensive set of experimental evaluations. Finally, Section 5 concludes the paper with conclusions, limitations, and directions for future work.

## 2. Related Work

Rubinstein et al. [22] propose a private kernel SVM algorithm (shortened as PrivateSVM) which only works for a translation-invariant kernel *g*(Δ). The method approximates the original infinite feature space *Ω* of *g*(Δ) with a finite feature space $\tilde{\Omega }$ using the Fourier transform *p*(*ω*) of *g*(Δ). Then add the noise to the weight parameters in the primal form based on the new space $\tilde{\Omega }$. One weakness is that the parameters used to construct $\tilde{\Omega }$ are randomly generated from *p*(*ω*) which degrades the approximation accuracy of $\tilde{\Omega }$ to *Ω*. Another problem is that the utility bounds use the same regularization parameter value to compare the private and nonprivate classifiers. They take no consideration into the change of regularization parameter incurred by privacy constraints. Chaudhuri et al. [23] investigated a general mechanism, namely, DPERM, to produce private approximations of classifiers by regularized empirical risk minimization (ERM) with good perturbation error. Akin to PrivateSVM, DPERM requires that the underlying kernel is translation invariant. In this paper, we will compare our method to the PrivateSVM algorithm, since DPERM has comparable performance with PrivateSVM.

## 3. Preliminary

Consider an original dataset *D* = {(**x**_*i*_, *y*_*i*_) | *i* ∈ *Z* ^+^,  1 ≤ *i* ≤ *n*} that contains a small portion of public data *D*_public_ and a large part of private data *D*_private_. Our goal is to release a differentially private support vector machine using both public and private data. In this section, we first introduce the definition of differential privacy; then, we give a brief overview of SVM and RBF kernel.

### 3.1. Differential Privacy

Differential privacy has emerged as one of the strongest privacy definitions for statistical data release. It guarantees that if an adversary knows complete information of all the tuples in *D* except one, the output of a differentially private randomized algorithm should not give the adversary too much additional information about the remaining tuples. We say that datasets *D* and *D*′ differ in only one tuple if we can obtain *D*′ by removing or adding only one tuple from *D*. A formal definition of differential privacy is given as follows.

**Definition (*ϵ*-differential privacy [18])**

Let *A* be a randomized algorithm over two datasets *D* and *D*′ differing in only one tuple, and let *O* be any arbitrary set of possible outputs of *A*. Algorithm *A* satisfies *ϵ*-differential privacy if and only if the following holds:

(1) Pr[A(D)∈O]≤eϵPr[A(D′)∈O].

Intuitively, differential privacy ensures that the released output distribution of *A* remains nearly the same whether or not an individual tuple is in the dataset.

A common mechanism to achieve differential privacy is the Laplace mechanism [18] that adds a small amount of independent noise to the output of a numeric function *f* to fulfill *ϵ*-differential privacy of releasing *f*, where the noise is drawn from* Laplace distribution* with a probability density function Pr[*η* = *x*] = (1/2*b*)*e* ^−|*x*|/*b*^. A Laplace noise has a variance 2*b* ^2^ with a magnitude of *b*. The magnitude *b* of the noise depends on the concept of* sensitivity* which is defined as follows.

**Definition 2 (sensitivity [18])**

Let *f* denote a numeric function, and the sensitivity of *f* is defined as the maximal *L*_1_-norm distance between the outputs of *f* over the two datasets *D* and *D*′ which differ in only one tuple. Formally,

(2) $$\begin{array}{c} \Delta_{f}=\underset{D,D^{\prime }}{\max}{||f(D)-f\left(D^{\prime }\right)||}_{1}. \end{array}$$

With the concept of sensitivity, the noise follows a zero-mean Laplace distribution with the magnitude *b* = Δ_*f*_/*ϵ*. To fulfill *ϵ*-differential privacy for a numeric function *f* over *D*, it is sufficient to publish *f*(*D*) + *X*, where *X* is drawn from Lap(Δ_*f*_/*ϵ*).

### 3.2. Review of SVM and RBF Kernel

SVM is one of the most popular supervised binary classification methods that takes a sample and a predetermined kernel function as input, and outputs a predicted class label for this sample. Consider training data *D* = {(**x**_*i*_, *y*_*i*_) | *i* ∈ *Z* ^+^,  1 ≤ *i* ≤ *n*}, where **x**_*i*_ ∈ *R* ^*d*^ denotes the training input points, *y*_*i*_ ∈ {1, −1} are the training class labels, and *n* is the size of training data. Here, *d* is the dimension of input data and “+1” and “−1” are class labels. A SVM maximizes the geometric margin between two classes of data and minimizes the error from misclassified data points. The primal form of a soft-margin SVM can be written as

(3) $$\begin{array}{c} \underset{w\in R^{F}}{\min}\frac{1}{2}{||w||}_{2}^{2}+C\overset{n}{\underset{i=1}{\sum }}l\left(y_{i},f_{w}\left(x_{i}\right)\right), \end{array}$$

where **w** is the normal vector to the hyperplane separating two classes of data, $l(y,\hat{y})$ is a loss function convex in $\hat{y}$, *C* is a regularization parameter that weighs smoothness and errors (i.e., large for fewer errors, smaller for increased smoothness), and *f*_**w**_(**x**_*i*_) = 〈*ϕ*(*x*_*i*_), **w**〉, where *ϕ*(**x**) : *R* ^*d*^ → *R* ^*F*^ is a function mapping training data point from their input space *R* ^*d*^ to a new *F*-dimensional feature space *R* ^*F*^ (*F* may be infinite). Sometimes we map the training data from their input space to another high-dimensional feature space in order to classify nonlinearly separable data. When *F* is large or infinite, the innerproducts in feature space *R* ^*F*^ may be computed efficiently by an explicit representation of the kernel function *k*(**x**, **y**) = 〈*ϕ*(**x**), *ϕ*(**y**)〉. For example, *k*(**x**, **y**) = **x** ^*T*^ **y** is a linear kernel function for a linear SVM, and *k*(**x**, **y**) = exp⁡(−||**x**−**y**||_2_^2^/*σ* ^2^) is a RBF kernel function, which is translation invariant.

In this paper, we use a RBF kernel function. Our method can be applied to any translation invariant kernel SVM. With the hinge loss *l*(*y*_*i*_, *f*_**w**_(**x**_*i*_)) = max⁡(0,1 − *y*_*i*_ *f*_**w**_(**x**_*i*_)), we can obtain a dual form SVM written as

(4) max⁡α∈Rn ∑i=1nαi−12∑i=1n ∑j=1nαiαjyiyjk(xi,xj)s.t. 0≤αi≤C, ∀i∈1,…,n,

where *α*_*i*_ ∈ **α**, *i* ∈ (1, *n*) is a persample parameter and *w*_*j*_ ∈ **w**, *j* ∈ (1, *d*) is a perfeature weight parameter. The weight vector **w** can be converted from sample weight vector **α** via **w** = ∑_*i*=1_^*n*^ *y*_*i*_ *α*_*i*_ **x**_*i*_ in the linear SVM.

## 4. Privacy Preserving Hybrid SVM

In this section, we first introduce a framework overview and then the technical details of our hybrid SVM method. We assume that all data samples follow the same distribution. Here, we assume that all original data from different data sets follow some unknown joint multivariate distribution and all data tuples are samples from this distribution.

### 4.1. The General Framework

**Figure 2:**
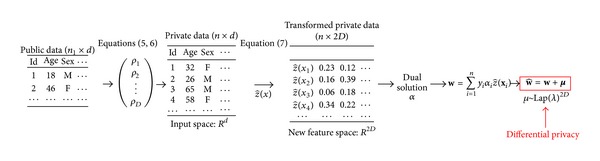
Detailed framework of our hybrid SVM.

Figure 2 illustrates the general framework of hybrid SVM.  Algorithm 1 presents the hybrid SVM algorithm. First, we use the small amount of public data and (5) and (6) to compute the parameter **ρ** = (**ρ**_1_,…, **ρ**_*D*_)^*T*^, **ρ**_*i*_ ∈ *R* ^*d*^ in the mapping function of the approximation form to the RBF kernel. Second, with **ρ**, we transform the private data from the original sample space to the new 2*D*-dimensional feature space via the mapping function $\hat{z}(x)$ in (7). Then we can compute the parameter **α** in the dual space with the transformed private data and **w** in the primal space via the linear relationship between **α** and **w** in the linear SVM. Finally, draw ***μ*** from Lap(*λ*)^2*D*^ where $\lambda =2^{2.5}C\sqrt{D}/n\epsilon$ and return $\hat{w}=w+\mu$ and **ρ**. Then users can transform their test data to the new 2*D*-dimensional feature space with **ρ** and classify the transformed data with $\hat{w}$. Here the computation of parameter **ρ** has no privacy risk because it is retrieved directly from public data. More details about hybrid SVM will be given in the successive subsections.

*Privacy Properties*. We present the following theorem showing the privacy property of Algorithm 1.

**Theorem 3**

Algorithm 1 guarantees *ϵ*-differential privacy.

**Proof**

For step 1, no private data is used, and hence step 1 does not impact the privacy guarantee. Due to Corollary 15 in [22] and the fact that the hinge-loss is convex and 1-Lipschitz in $\hat{y}$, the sensitivity of **w** over a pair of neighbouring datasets is $\Delta_{w}=2^{2.5}C\sqrt{D}/n$. Then the scale parameter *λ* in step 4 is set to $\lambda =\Delta_{w}/\epsilon =2^{2.5}C\sqrt{D}/n\epsilon$ due to the Laplace mechanism introduced in Section 3.1. Therefore, Algorithm 1 preserves *ϵ*-differential privacy which completes the proof.

### 4.2. The Computation of **ρ**

Rahimi and Recht [24] approximate a Reproducing Kernel Hilbert Space (RKHS) *H* induced by an infinite dimensional feature mapping with a random $\text{RKHS}\hat{H}$ induced by a random finite-dimensional mapping *z*. The random finite-dimensional $\text{RKHS}\hat{H}$ can be constructed by drawing *D* i.i.d. vectors **ρ**_1_,…, **ρ**_*D*_ from the Fourier transform of a positive-definite translation-invariant kernel function *k*(*x*, *y*), such as the RBF kernel function. Then we can obtain an approximation form *z*(*x*)^*T*^ *z*(*y*) of *k*(*x*, *y*) using the real-valued mapping function *z*(*x*) : *R* ^*d*^ → *R* ^*D*^ defined by the following equation:

(5) $$\begin{array}{c} z\left(x\right)=\sqrt{\frac{2}{D}}{\left[\cos\left(\rho_{1}^{T}x+b_{1}\right)\cdots \cos\left(\rho_{D}^{T}x+b_{D}\right)\right]}^{T}, \end{array}$$

where *b*_1_,…, *b*_*D*_ are i.i.d. samples drawn from a uniform distribution *U*[0,2*π*]. *z*(*x*) : *R* ^*d*^ → *R* ^*D*^ maps the data from its original *d*-dimensional input space to the new *D*-dimensional feature space. Their approach is based on the fact that the kernel function of a continuous positive-definite translation-invariant kernel is the Fourier transform of a nonnegative measure. The uniform convergence property of the approximation form *z*(*x*)^*T*^ *z*(*y*) to the kernel function *k*(*x*, *y*) has also been proved in [24]. In our context, the kernel function *k*(*x*, *y*) refers to the RBF kernel function.

In our problem setting, since a small amount of public data can be considered as *x* in *z*(*x*) and only the vectors **ρ**_1_,…, **ρ**_*D*_ are needed to construct the random finite-dimensional $\text{RKHS}\hat{H}$, we can compute the vectors **ρ**_1_,…, **ρ**_*D*_ with an optimization function defined as follows:

(6) $$\begin{array}{c} \underset{\rho \in R^{D\times d}}{\min}\overset{n}{\underset{i=1}{\sum }}\;\overset{n}{\underset{j=1}{\sum }}\left|\frac{2}{D}z{\left(x_{i}\right)}^{T}z\left(x_{j}\right)-k\left(x_{i},x_{j}\right)\right|. \end{array}$$

Since (6) is an unconstrained nonlinear optimization function, we solve it using L-BFGS (the full name is Limited-memory Broyden Fletcher Goldfarb Shanno) algorithm.

Thus, we can obtain a more accurate approximation form *z*(*x*)^*T*^ *z*(*y*) of the kernel function *k*(*x*, *y*) by deploying the public data to compute the **ρ**, than randomly sampling **ρ** from the fourier transform of the kernel function *k*(*x*, *y*) as shown in [25]. To guarantee differential privacy, we need only consider the data-dependent weight parameter **w**. Fortunately we can employ the differentially private linear SVM approach in [25] to compute **w** after transforming all private data to a new 2*D*-dimensional feature space using the mapping $\hat{z}(x):R^{d}\to R^{2D}$ defined in (7) with the vectors **ρ**_1_,…, **ρ**_*D*_ as follows:

(7) z^(x)=1D[cos⁡⁡(ρ1Tx),sin⁡(ρ1Tx),…,    cos⁡(ρDTx),sin(ρDTx)]T.

### 4.3. The Computation of **$\hat{w}$**

With the vectors **ρ**_1_,…, **ρ**_*D*_ to approximate the RBF kernel function, we can convert RBF kernel SVM in the *d*-dimensional input space into the linear SVM in a new 2*D*-dimensional feature space with (7), then use the privacy preserving linear SVM algorithm in [25]. The general idea of this algorithm is that with the transformed 2*D*-dimensional private data, we first compute the parameter **α** in the dual space and then **w** in the primal space using $w=\sum_{i=1}^{n}y_{i}\alpha_{i}\hat{z}(x_{i})$; then we draw ***μ*** from Lap(*λ*)^2*D*^, where $\lambda =2^{2.5}C\sqrt{D}/n\epsilon$ and compute noisy $\hat{w}$ with $\hat{w}=w+\mu$.

## 5. Experiments

In this section, we experimentally evaluate our hybrid SVM and compare it with one state-of-the-art method, called private SVM and on baseline method. We evaluate the utility of the trained SVM classifier using the AUC metric. Hybrid SVM and private SVM are implemented in MATLAB R2010b, and all experiments were performed on a PC with 3.2 GHz CPU and 8 G RAM.

### 5.1. Experiment Setup

*Datasets*. We used two open source datasets from the Integrated Public Use Microdata Series (Minnesota Population Center, Integrated public use microdata series—international: Version 5.0., 2009, https://international.ipums.org),   the US and Brazil census datasets with 370,000 and 190,000 records collected in the US and Brazil, respectively. One motivation for using these public datasets is that it bears similar attributes (e.g., demographic features) as some medical records, but it is publicly available for testing and comparisons. From each dataset, we selected 40,000 records, with 10,000 records serving as the public data pool. There were 13 attributes in both datasets, namely,* age, gender, marital status, education, disability, nationality, working hours per week, number of years residing in the current location, ownership of dwelling, family size, number of children, number of automobiles*, and* annual income*. Among these attributes,* marital status* is the only categorical attribute containing more than 2 values, that is,* single*,* married*, and* divorced/widowed*. Because SVMs do not handle categorical features by default, we transformed* marital status* into two binary attributes,* is single* and* is married* (an individual divorced or widowed would have false on both of these attributes). With this transformation, our two datasets had 14 dimensions. For each dataset, we randomly extract a subset of original data as a public data pool, from which public data is sampled uniformly, and use the remaining 30000 tuples as the private data.

*Comparison.* We experimentally compared the performance of our hybrid SVM against two approaches, namely, public data baseline and private SVM [25]. The public data baseline is a RBF kernel SVM that uses only public data. In our experiment figures, we use “Public—#” to denote the public data baseline method with # as the size of public data. The private SVM is a state-of-the-art differentially private RBF kernel SVM that uses private data only. The parameters in all methods are set to optimal values.

*Metrics.* We used the other attributes to predict the value of* annual income* by converting* annual income* into a binary attribute: values higher than a predefined threshold were mapped to 1, and otherwise to −1. Here, we set the predefined threshold as the median value of* annual income*. The classification accuracy was measured by the AUC (the area under an ROC curve) [26]. The boxplot was used to measure the stability of our method and private SVM. The boxplots of “Public—50,” “Public—100,” and “Public—200,” are qualitatively similar to our hybrid SVM; hence, we do not report boxplots of these baseline methods. We performed 10-fold cross-validation 10 times for each algorithm and reported the average results. We varied three different parameters: the privacy budget *ϵ*, the dataset dimensionality, and the data cardinality (i.e., the size of training data). To vary the data cardinality parameter, we randomly generate subsets of records in the training records set, with the sampling rate varying from 0.1 to 1. For various data dimensionalities with the range being 5, 8, 11, and 14, we select three attribute subsets in the US and Brazil datasets for classification. The first five dimensions include:* age*,* gender*,* education*,* family size*, and* annual income*. The second eight dimensions contain the previous five attributes, and additionally* nativity*,* owner of dwelling*, and* number of automobiles*. The third eleven dimensions consist of all the attributes in the second 8 dimensions and* is single*,* is married*, and* number of children*.  Table 1 summarizes the parameters and their default values in the experiments.

### 5.2. AUC versus Privacy Budget

**Figure 3:**
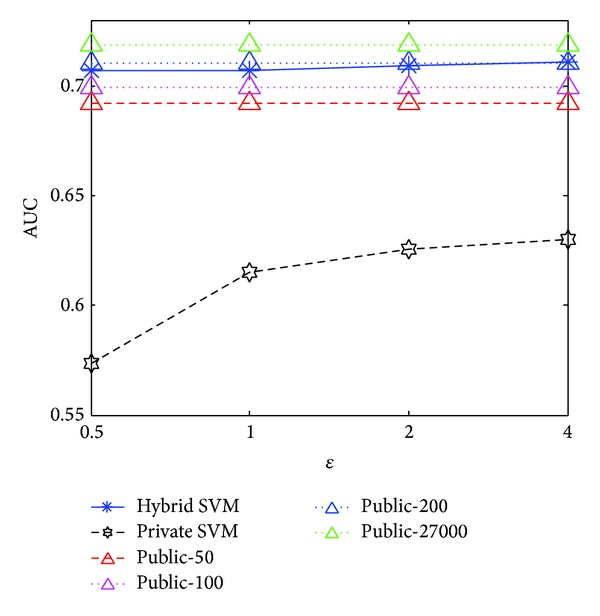
AUC versus privacy budget for US.

**Figure 4:**
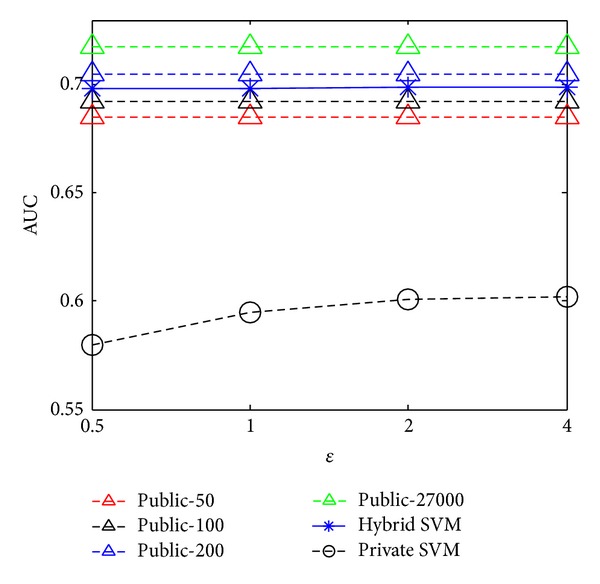
AUC versus privacy budget for Brazil.

Figures 3 and 4 illustrate the AUCs of each method under various privacy budgets from 0.5 to 4, where “Public—#” means the public data baseline methods with various sizes of public data. Observe that our hybrid SVM outperforms the private SVM and performs better than the public data baseline defined by the public data. The AUC of our method remains stable under all privacy budgets and is significantly close to the public data baseline that uses the complete private data set as public data.

### 5.3. AUC versus Dataset Dimensionality

**Figure 5:**
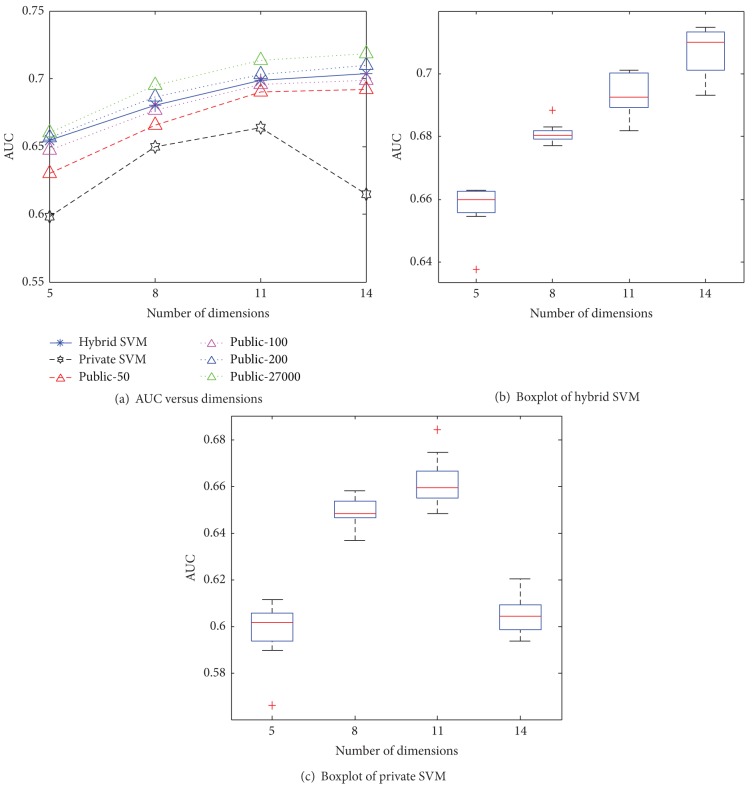
AUC versus dimensions for US.

**Figure 6:**
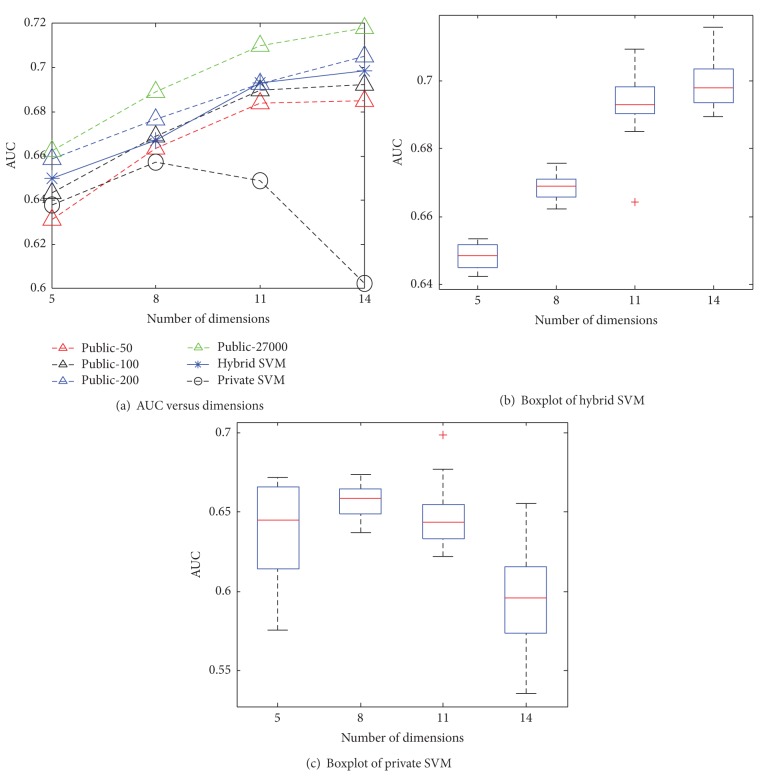
AUC versus dimensions for Brazil.

Figures 5 and 6 present the AUCs of each algorithm as a function of the dataset dimensionality for the US and Brazil datasets. With a higher number of dimensions, the AUCs of the hybrid SVM and of the SVM that uses the public data (baseline) increase. This is reasonable because the training data size with the default value being 27,000 is much larger than the number of data dimensions which are at most 14. When the number of dimensions grows, the performance improves. In contrast, the performance of the private SVM degrades in 14 dimensions with poor boxplots because more noise is introduced with higher dimensions.

### 5.4. AUC versus Data Cardinality

**Figure 7:**
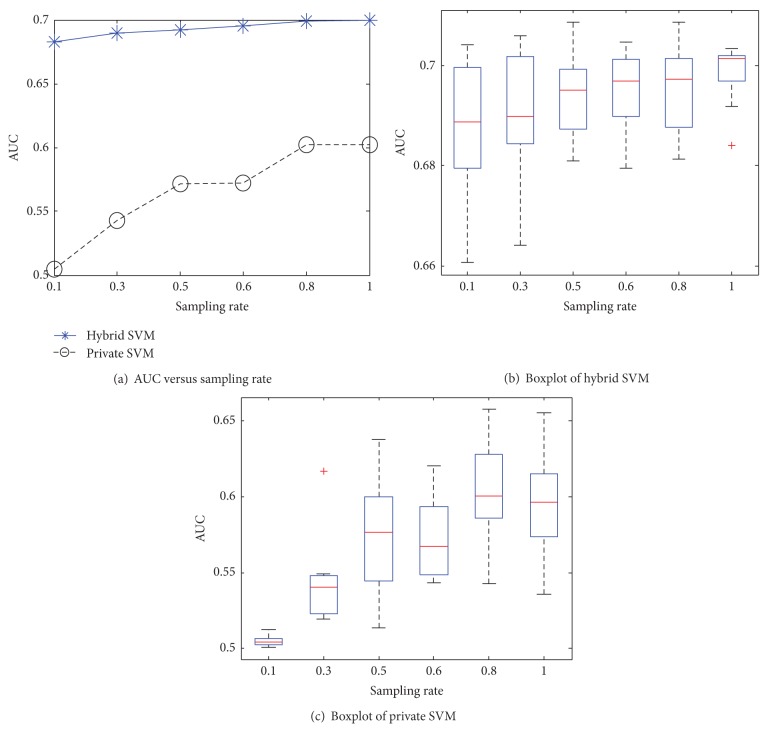
AUC versus sampling rate for US.

**Figure 8:**
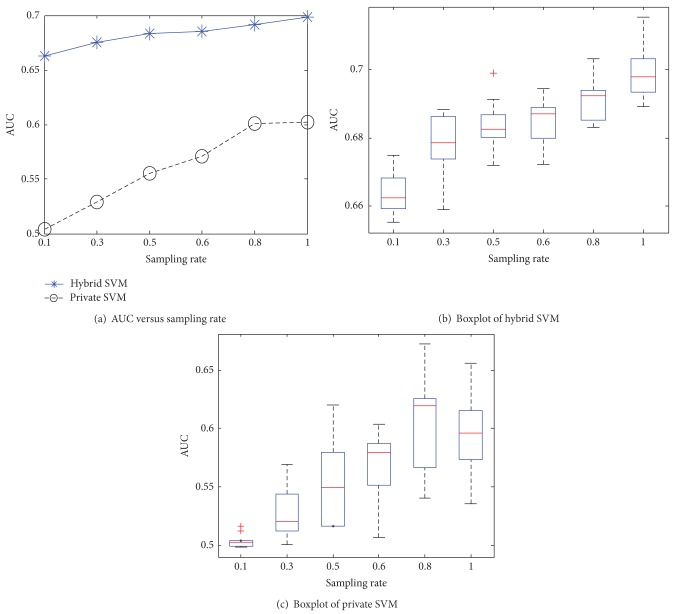
AUC versus sampling rate for Brazil.

Figures 7 and 8 investigate the relationship between the sampling rate and AUC of hybrid and private SVMs. From the figures, our method consistently outperformed the private SVM at different sampling rates. It is worth mentioning that AUCs of the hybrid SVM are large even at small sampling rates and tend to stabilize when the size of training data grows (i.e., large sampling rate). The boxplots reflect that the private SVM has larger variance than the hybrid SVM, because private SVM selects the values of **ρ** randomly from the Fourier transform of RBF kernel. In contrast, hybrid SVM computes **ρ** via the public data. This helps improve the accuracy of **ρ** and leads to less variance.

### 5.5. Computation Time

**Figure 9:**
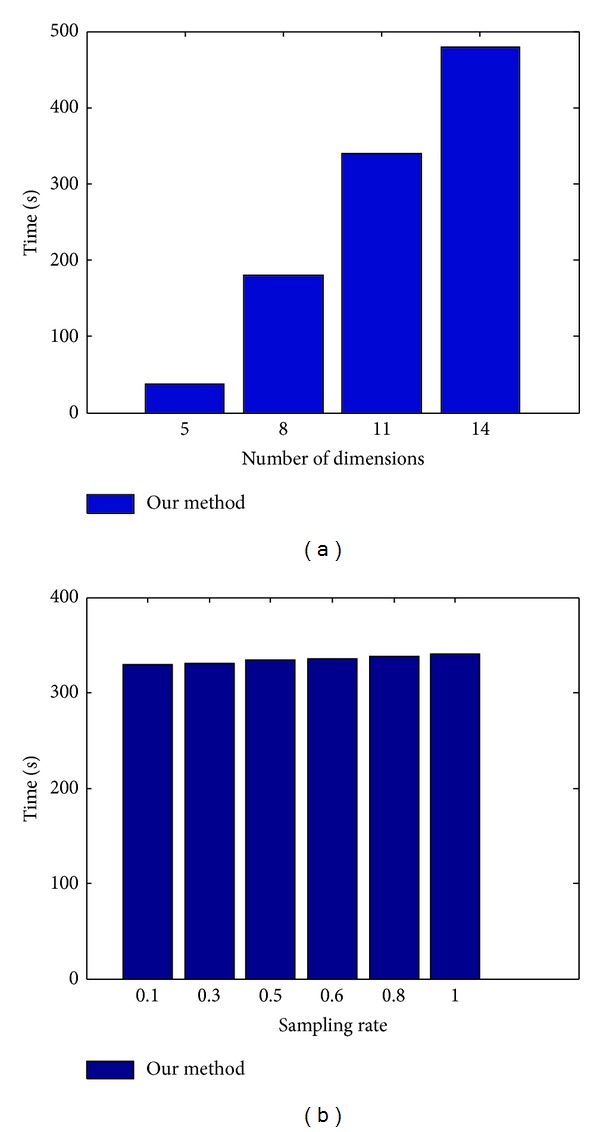
Time versus dimensions and sampling rate.

Finally, Figure 9 shows the time cost of our proposed algorithm with varying dimensions and different sampling rates. We only report the results for the US dataset; the results for the Brazil dataset are greatly similar. One can notice that the dimensionality, rather than the sampling rate, determines the computational cost of the hybrid SVM. The overhead of the hybrid SVM is from computing **ρ** with the public data, since a nonlinear optimization equation needs to be solved. As the other private SVM methods, our hybrid SVM is intended for off-line use, and hence the time is generally acceptable for even 14 dimensional datasets.

## 6. Discussion and Conclusion

We proposed and developed a RBF kernel SVM using a small amount of public data and a large amount of private data to preserve differential privacy with improved utility. In this algorithm, we use public data to compute the parameters in an approximation form of the RBF kernel function and then train private classifiers with linear SVM after converting all private data into a new feature space defined by the approximation form. A limitation of our approach is that we used the L-BFGS method [27], which is not very efficient, to find the optimal solution. Because the objective function in (6) is not a convex function, our model is computationally intensive in order to calculate the local optimal values, especially when the size of the public data set is large. We will develop more efficient methods and test the model on clinical records in future work. Another limitation is that we assume all original data from different data sets follow some unknown joint multivariate distribution. Our assumption might now always be true in practice, and calibration is necessary for future investigation. That is, in the presence of distributional difference, we will leverage transfer learning to build the global model.

## Figures and Tables

**Algorithm 1:**
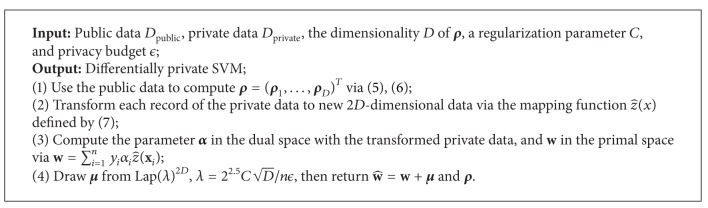
Hybrid SVM algorithm.

**Table 1:** Experiment parameters.

Parameter	Default value
Number of records in the public data used by hybrid SVM	20
Number of records in the private training dataset	27000
Number of records in the test dataset	3000
Number of dimensions	14
Privacy budget *ϵ*	1.0
